# Design of UAV Downwash Airflow Field Detection System Based on Strain Effect Principle

**DOI:** 10.3390/s19112630

**Published:** 2019-06-10

**Authors:** Yalei Wu, Lijun Qi, Hao Zhang, Elizabeth M. Musiu, Zepeng Yang, Pei Wang

**Affiliations:** 1 College of Engineering, China Agricultural University, No.17 Qing Hua Dong Lu, Haidian District, Beijing 100083, China; kevin_wuyalei@cau.edu.cn (Y.W.); zhanghao08@cau.edu.cn (H.Z.); yzp3619@cau.edu.cn (Z.Y.); 2 Agricultural and Biosystem Engineering Department, Jomo Kenyatta University of Agriculture and Technology, Nairobi P.O Box 62000-00200, Kenya; musiu@jkuat.ac.ke; 3 Key Laboratory of Modern Agricultural Equipment and Technology, Ministry of Education of PRC, Jiangsu University, Zhenjiang 212300, China; wangpei@live.cn

**Keywords:** strain effect principle, flexible polypropylene detection structure, ANSYS simulation, one-way fluid-solid coupling, downwash airflow wind field measurement

## Abstract

Accurate measurement of the downwash flow field of plant protection unmanned aerial vehicles (UAVs) is essential for analyzing the spatial distribution of droplets. To realize on-line rapid detection of the downwash flow field of a multi-rotor UAV, a flexible polypropylene detection device based on the principle of full bridge strain effect was proposed. Its performance principle was based on the physical deformation caused by wind pressure. The Fluid Flow and Static Structural modules of ANSYS 16.0 finite element software were used to simulate one-way fluid-solid coupling interaction. The surface of the resistive strain gauge embedded in the flexible detecting structure responded well to wind speed variation, hence it was suitable for downwash airflow wind field detection. By solving the strain force on the surface of the flexible detection structure, the length and layout of the grating wire of the strain gauge on the surface of the flexible detection structure were optimized. Meanwhile at 4 m·s^−1^ wind speed, the output voltage at varied bridge flexible acquisition systems in the acquisition card was measured. Results indicated coefficient of variation of 3.67%, 1.63% and 1.5%, respectively, which proved the good data acquisition consistency of the system. Through calibration test, the regression equation for the relationship between output voltage and wind speed for three unique sensor signal measuring circuits was established. The determination coefficients *R*^2^ for single bridge, half bridge and full bridge circuits were 0.9885, 0.9866 and 0.9959, respectively. In conclusion, by applying the multi-rotor plant protection UAV test platform, the results indicated the maximum relative error of the wind speed at each sampling point of the system at 1.0 m altitude was below 5.61%. Simulated and measured value had an *RMSE* maximum error of 0.1246 m·s^−1^. Moreover, downwash airflow detection not only has high accuracy but also has high sensitivity. Thus, there is convenience and practicability in the plant protection offered by this approach. The rapid measurement of UAV wind field and the established two-dimensional wind field model can provide a basis for precise application of agricultural aviation.

## 1. Introduction

The multi-rotor unmanned aerial vehicle (UAV) has the advantages of high efficiency, strong droplet penetration, no need for a dedicated lifting platform, and good maneuverability. Its application in crop protection has rapidly grown in recent years [1,2,3]. When the UAV is used for spraying, the airborne spraying equipment converts the liquid into droplets but leaves the fuselage. At this point, the downwash flow plays an important role especially in defining how the droplets attach to the crop canopy. Additionally, the contact area of the downwash flow acting on the crop canopy establishes the target area of spraying droplets, which in essence determines the deposition location of droplets. Meanwhile, the wind power of downwash flow directly affects the penetration and deposition rate of droplets [4]. Previous studies have shown that the downwash flow field of multi-rotor plant protection UAV directly determines the movement and diffusion of droplets in space [5,6]. Fast and effective acquisition of the distribution characteristics and rules of the downwash flow field does not only capture the parameters of space wind field in time, but also has a significant impact on the evaluation of penetration [7], deposition distribution of droplets and the improvement of pesticide utilization rate [8,9]. The optimization of detection technology of downwash airflow field distribution of multi-rotor plant protection UAV to curb the high cost, low efficiency and poor accuracy of field wind field measurement has become a hot research topic in this field [10].

At present, wind measurement methods mainly include the mechanical [11], Pitot tube [12], hot wire [13], heat sensitive [14], and ultrasonic types [15]. The mechanical impeller anemometer is the most widely used piece of equipment for measuring the wind speed of domestic agricultural UAV. This device mainly relies on the downwash air of UAV to rotate the impeller and thus generate electromagnetic signals, which in return give feedback on wind speed [16]. The advantages of this type of sensor are that they are durable and give long-term measurements. However, the impeller anemometers are voluminous, have larger interference to the wind field, and poor sensitivity. The Pitot tube wind field testing device has small disturbance and high sensitivity [17], but it requires a homogenous flow field. Logically, it is easy for this device to give inaccurate measurements, especially in environment with a heterogeneous flow field. The hot-wire film type and the ultrasonic type are also used in wind speed measurement. Based on the principle of ultrasonic time difference wind measurement, Zhang et al. [18] evaluated the shadow effect compensation. Their approach incorporated computational fluid dynamics (CFD) simulation and back propagation (BP) neural network data processing. Mullins et al. [19] introduced a method to realize a hot-wire film integrated silicon anemometer. The new technology uses an integrated temperature-insensitive operational amplifier to realize thermal feedback in the double Wheatstone bridge structure, which is superior to the mechanical peak flowmeter. Compared to the mechanical measurement method, the hot-wire film type and the ultrasonic type have relatively few applications in wind speed measurement. In addition, in order to realize the on-line rapid detection of wind field, previous researchers [20,21,22] designed a wind field wireless sensor network measurement system. The reliability of their system in wind speed measurement was analyzed via field tests. Although, the law of wind field distribution was obtained after analyzing the collected wind speed data, the two-dimensional shape of the rotor downwash airflow field after incorporation of the crop canopy was not directly reflected. In general, most of the existing wind field testing equipment for agricultural UAV are built based on the principle of the mechanical impeller [17]. The impeller anemometer has a large shape and has a large interference to the wind field measurement which affects the accuracy of the wind speed measurement. Since the impeller blades have inertia, the perception of wind speed changes is poor, hence reaction is delayed, and the response is slow. In addition, there are relatively few devices that enable real-time monitoring in conjunction with sensor network technology. Although literature on wind speed measuring equipment, methods, and online rapid detection [23] exists, the detection of the downwash airflow field under UAV has not fully been elucidated.

Based on the principle of the strain effect of full bridge and sensor network technology, the objective of this paper was to design a real-time detection system for the downwash airflow field of multi-rotor UAV. CFD simulation was used as a means to carry out one-way fluid-solid coupling simulation analysis [24], and the grating length and arrangement form of strain gauges on the surface of flexible target structure are optimized step by step. At the same time, the calibrated grating length and arrangement form of strain gauges were combined with the multi-rotor UAV system platform. The wind field model was subjected to tests and verified in order to provide reference for obtaining the distribution characteristics of downwash airflow field accurately and quickly on-line.

## 2. Measurement Principle of Acquisition System

**Figure 1:**
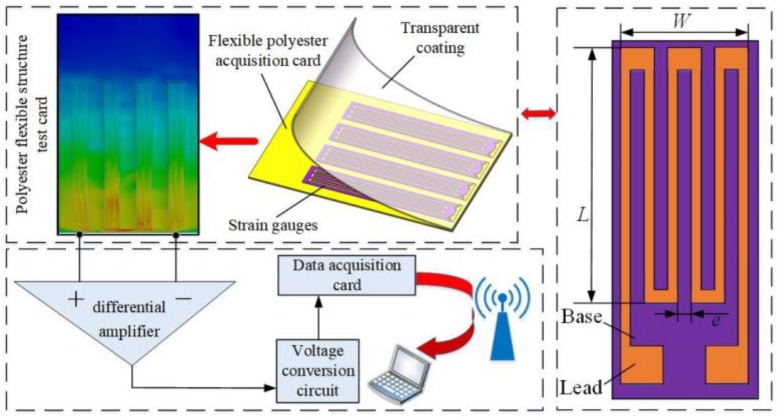
Schematic diagram of real-time detection device system for an unmanned aerial vehicle (UAV). Note: *W* is the width of the grating wire; *L* is the length of the grating wire; *e* is the spacing of the grating wire.

The downwash airflow field flexible detection structure acquisition system was designed based on the principle of the strain effect as previously applied by Biswal and Ramaswamy [25]. It consisted of a polypropylene flexible detection structure acquisition card; aluminums bracket support frame; DC power supply; integrated circuit control board; strain gauge full bridge circuit; differential amplifier; EM9636M data acquisition card; wireless network transmission device; and PC terminal. Data storage, processing, calibration and model calibration were performed in the LabVIEW 2014 host computer acquisition interface. The full bridge strain effect was used to measure the UAV downwash airflow field, which actually reflects the characteristic impedance change of strain gauge in the flexible detection structure acquisition system under the pesticide application environment. The proposed system has the advantage of wind pressure conversion, and the sensitivity is high. Figure 1 shows the schematic diagram of the UAV downwash airflow field real-time detection device system.

The strain gauge was glued on the surface of the flexible detection structure acquisition card and covered with the transparent coating layer. Ideally, when the downwash airflow field generated by the UAV interacts with the flexible detection structure acquisition card [26], the flexible detecting structure deform due to the wind pressure, and the strain gauge passively deform correspondingly. The resistance value thereof changes, and finally the voltage amplitude at the test point changes. Thus, through the change of the voltage amplitude, the change of the resistance is indirectly inversed, and then the wind speed of the downwash airflow field is obtained [27,28].

The strain gauge used was a piezoresistive strain, which in essence is the principle of strain effect. When the strain gauge is deformed by the action of wind pressure, the relationship between the amount of change in electrical resistance and the deformation is satisfied according to Equation (1) as:

(1) $$\frac{\Delta R}{R}=(1+2\mu )\frac{\Delta l}{l}+\frac{\Delta \rho }{\rho }≃(1+2\mu )\varepsilon_{0},$$

where, ΔR/R is the relative change amount of the strain gauge resistance; $\varepsilon_{0}=\Delta l/l$ is the relative deformation amount of the strain gauge; μ is the Poisson’s ratio of the strain gauge material; Δρ/ρ is the relative change amount of the strain gauge resistivity, which is small and negligible.

**Figure 2:**
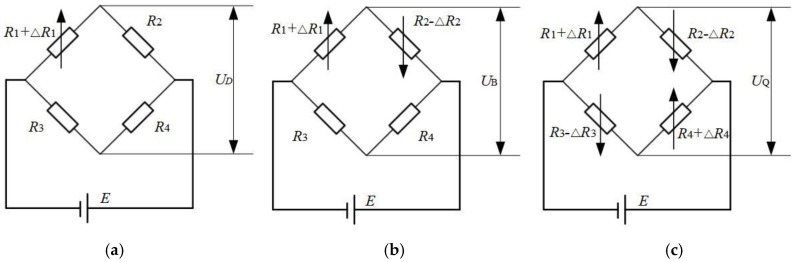
Measuring device equivalent circuit diagram. (**a**) Single bridge circuit; (**b**) Half bridge circuit; (**c**) Full bridge circuit.

According to the piezoresistive strain theory [25], and Equation (1), the value of ΔR/R is usually small, thus the sensitivity of the measured strain gauge is weak. Therefore, this study opted to use a single-bridge circuit, a differential half-bridge circuit and a differential full-bridge circuit in order to improve the measurement sensitivity of the system. The three types of bridges were considered because different numbers of strain gauge perform differently. Moreover, the use of different bridges allowed readers to have a more comprehensive grasp of the performance characteristics of the sensor. This therefore can lay a foundation for the subsequent optimization of the design of flexible detection structure acquisition card system. Figure 2 shows the equivalent circuit diagram. 

In the measuring circuit of Figure 2a,c, the precondition for the bridge to be at equilibrium is that the strain gauge is not subjected to external pressure, and the product of resistance of the corresponding arm of the bridge is equal.

In this paper, the performance of the differential half-bridge circuit of Figure 2b was first analyzed. Presumably when the strain gauge is deformed by wind pressure $\Delta R_{1}=\Delta R_{2}\neq 0$, at this point the bridge is no longer at equilibrium, hence $U_{B}$ changes according to the expression:

(2) $$U_{B}=E(\frac{R_{1}+\Delta R_{1}}{R_{1}+\Delta R_{1}+R_{2}-\Delta R_{2}}-\frac{R_{3}}{R_{3}+R_{4}}),$$

Assuming $\lambda ≜R_{2}/R_{1}$, $\Delta R_{1}$ and $\Delta R_{2}$ are much smaller than $R_{1}$ and $R_{2}$. Combining with the condition of bridge balance $R_{1}R_{4}=R_{2}R_{3}$, the differential half bridge circuit voltage measurement sensitivity is obtained as follows:

(3) $$K_{B}=\frac{U_{B}}{\left(\Delta R_{1}/R_{1}\right)}=\frac{E}{\left(1+\lambda \right)},$$

Similarly, the sensitivity of strain gauges for single bridge and full bridge can be obtained according to the expression:

(4) $$K_{D}=\frac{E\lambda }{{\left(1+\lambda \right)}^{2}},$$

(5) $$K_{Q}=E,$$

where, $K_{D}$, $K_{B}$, $K_{Q}$ are the voltage measurement sensitivity of the single bridge, half bridge and full bridge, respectively; $R_{1}$, $R_{2}$, $R_{3}$, $R_{4}$ are the corresponding arm resistance value of the bridge; $U_{D}$, $U_{B}$, $U_{Q}$ are the output voltage value of the single bridge, half bridge, full bridge circuit, respectively.

In order to obtain the highest voltage output measurement sensitivity of the bridge measurement circuit, the first-order derivation of the single-bridge sensitivity Equation (4) was performed. To this effect: $\partial K_{D}/\partial \lambda =0$:λ=1. From the results of the derivation, it is known that the resistance value of the bridge arm is $R_{1}=R_{2}=R_{3}=R_{4}$, and the sensitivity is the highest [29]. At this premise, the sensitivity of the bridge from large to small is full bridge > half bridge > single bridge.

Based on the above analysis of bridge voltage sensitivity, it was theoretically determined that the full bridge had the highest voltage measurement sensitivity. At the same time, the differential measuring circuit had a temperature compensation function, and the measurement error was the smallest. In this paper, the strain-effect principle of the full-bridge measurement circuit was used to establish the relationship between the output voltage signal the wind speed measured by UAV downwash airflow field.

It has been shown by a previous study [30] that the larger the load applied to the surface of the object, the more the deformation. In this paper, the flexible detection structure acquisition card was selected as the blade target simulation material, and the polyester material had hysteresis. The deformation curve of the acquisition piece was slightly inconsistent before the wind pressure was applied and after the wind pressure was unloaded. In theory, if the stress-strain relationship of the material obeys Hooke’s law, and the relationship between wind pressure and the deformation of the flexible detection structure is given as:

(6) $$W_{P}=E^{*}L_{v},$$

where, $E^{*}$ is the calibration coefficient of the deformation model, and $L_{v}$ is the strain in the vertical direction of the acquisition card of the polyester flexible detection structure, m.

The pressure of downwash airflow field on the surface perpendicular to the direction of airflow is wind pressure. According to the Bernoulli principle, the relationship between the wind and the pressure is given as:

(7) $$W_{P}=\frac{r_{0}}{2}v^{2},$$

where, $W_{P}$ is the wind pressure, kN/m^2^, $r_{0}$ is the air density, kg/m^3^, v is the downwash field wind speed, m·s^−1^.

Given that the strain gauge was embedded in the flexible detection structure acquisition card, and the relative shape variable of the strain gauge had a good correlation with the relative deformation amount in the vertical direction of the flexible detection structure acquisition card, the correlation coefficient $K^{*}$ is expressed as:

(8) $$\frac{\Delta l}{l}=K^{*}\frac{\Delta L_{v}}{L_{v}},$$

Equation (2) establishes the relationship between output voltage and resistance change. At the same time, it is clear from Equation (1) that the resistance of strain gauge varies with respect to its deformation. Meanwhile, the deformation of the strain gauge varies with the deformation of the acquisition card of flexible detection structure. Therefore, the model correlation between the output voltage and the airflow velocity is deduced. The theoretical expression of the relationship between the change of voltage value and airflow velocity before and after testing the UAV downwash airflow field with full bridge sensing signal measuring circuit is as follows:

(9) $$U_{Q}=\frac{2EE^{*}K^{*}(1+2\mu )}{r_{0}v^{2}}\Delta (\frac{r_{0}v^{2}}{2E^{*}}),$$

Therefore, based on Equations (1)–(8), the variation of the characteristic airflow impedance of the strain gauge in the flexible detection structure acquisition system under the application environment can be used to characterize the change of the downwash airflow field. From theoretical analysis, the output voltage of the full bridge measuring circuit in the strain effect theory can be used to reflect the airflow velocity change of the downwash airflow field before and after the UAV test in the flexible detection structure acquisition system.

## 3. Strain Simulation Analysis and Parameter Determination of Acquisition System

Choosing the appropriate strain gauge structure parameters is vital in designing a flexible detection structure acquisition card system. The structural parameters have a great influence on the measurement accuracy of the sensor. In this paper, ANSYS 16.0 numerical simulation software was used to simulate the flexible detection structure acquisition system. The purpose was to find the optimal structural parameters of the strain gauge and provide a basis for the structural design of the flexible detection structure acquisition card and the selection of the strain gauge.

### 3.1. Finite Element Modeling and One-Way Fluid-Solid Coupling Analysis

#### 3.1.1. Establishment of Rotor Finite Element Model

**Figure 3:**
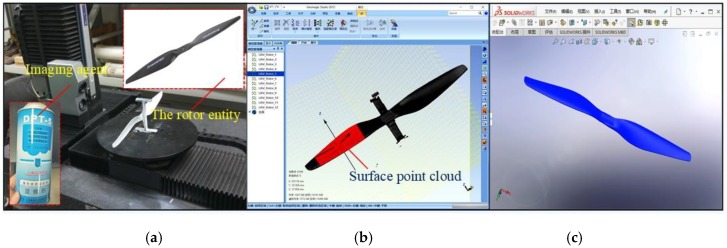
Rotor reverse modeling process. (**a**) Rotor surface scanning; (**b**) Geomagic Studio post processing of point cloud data; (**c**) Solidworks reconstruction rotor diagram.

The rotor is the key model component for simulation analysis. Based on reverse engineering technology, this paper used an automatic three-dimensional CaMega optical scanning system (MCS-5/4-axis, BoWeiHengXin, China) to scan the front and back rotor surfaces to obtain surface point cloud data. Figure 3a shows the set up for scanning of the rotor surface. The team used Geomagic Studio software to post-process the point cloud data (Figure 3b) to realize the reverse reconstruction of the rotor model (Figure 3c). This approach was previously applied by Zhang [27]. Then an accurate three-dimensional model of the rotor was obtained, which was used for subsequent one-way fluid-structure coupling simulation analysis.

The selection of the grating wire parameters is vital in establishing the acquisition card model of flexible detection structure. Previous research [31] shows that the length *L*, width *W* and spacing *e* of the grating wire have influence on the strain transfer error, and the length *L* of the grating wire has the greatest influence. Considering that the length of the polyester card is 80 mm, the number of tests required for all experiments is too large and at the same time it is limited by the type of the strain gauge. Notwithstanding, three representative grating wire lengths (*L* = 20 mm, 50 mm, 80 mm) in the length range were selected. Polypropylene was selected as the material of the acquisition card: geometric size (length × width × height) was 80 mm × 50 mm × 0.8 mm, modulus of elasticity was 896 N/mm^2^, Poisson’s ratio was 0.4103.

#### 3.1.2. Regional Boundary Setting and One-Way Fluid-Solid Coupling Analysis 

**Figure 4:**
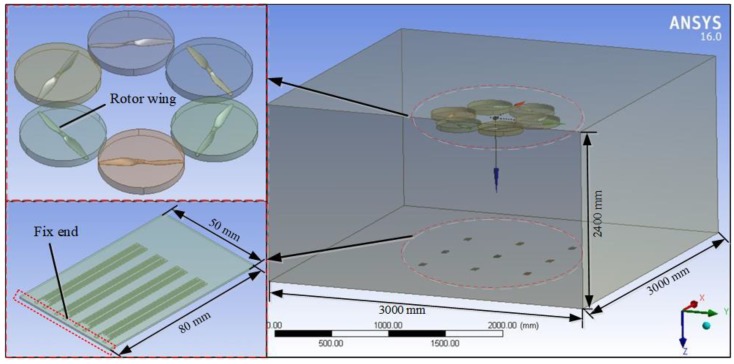
One-way fluid-solid coupling field modeling.

In terms of setting the boundary conditions (BC) of the area, the geometry of the calculation area was a cuboid with a length, width and height of 3 m × 3 m × 2.4 m, respectively. The computational domain consists of a cuboid domain and a cylindrical rotation domain (Figure 4). In the Fluid Flow module, the interface between the cube domain and a cylindrical rotation domain was connected through an interface. The rotor wall was set to the wall boundary condition, and the rest of the exit was set at pressure-outlet BC. Then, the cylindrical rotation domain was processed by the Multiple Reference Frame (MRF) model, and the steady state was set to solve. The Reynolds Averaged Navier-Stokes (RANS) equation of the plant protection UAV rotor airflow field was used as the basic control equation.

From a one-way and two-way data transmission point of view, fluid-structure coupling can be divided into one-way and two-way fluid-structure coupling. One-way fluid-solid coupling refers to the transfer of the results of CFD analysis (wind pressure load) to solid structure analysis. However, the deformation results of solid structure analysis in this study were so small that the influence on fluid analysis was neglected. On the other hand, the two-way fluid-structure coupling mainly solved the problem of large deformation. In this paper, the deformations of the flexible detection structure acquisition card under wind pressure were small (negligible effect of deformation on flow field distribution), which is common to most coupling phenomena. Only the static structure performance analysis was considered, so the one-way coupling analysis was adopted. In the ANSYS 16 one-way fluid-structure coupling analysis, the flow field of UAV downwash was first solved in Fluent, and then the pressure of the airflow field was applied as a load to the flexible detection structure acquisition card in Mechanical.

The acquisition card model of flexible detection structure was set as shown in Figure 4. Firstly, the fluid area was suppressed in the Static Structural module, and then the grating encryption divided corresponding to the position where the strain gauges was located, and the fluid load was introduced in the Mechanical module. Meanwhile, the unilateral side of the flexible detection structure was set as a fixed constraint (the red identification area in the lower left corner of Figure 4). Finally, the structural calculation was carried out, and the stress distribution and deformation of the flexible detection structure acquisition card were obtained.

### 3.2. Acquisition System Simulation Analysis and Parameter Determination

#### 3.2.1. Simulation of the Downwash Airflow Field under the Rotor

**Figure 5:**
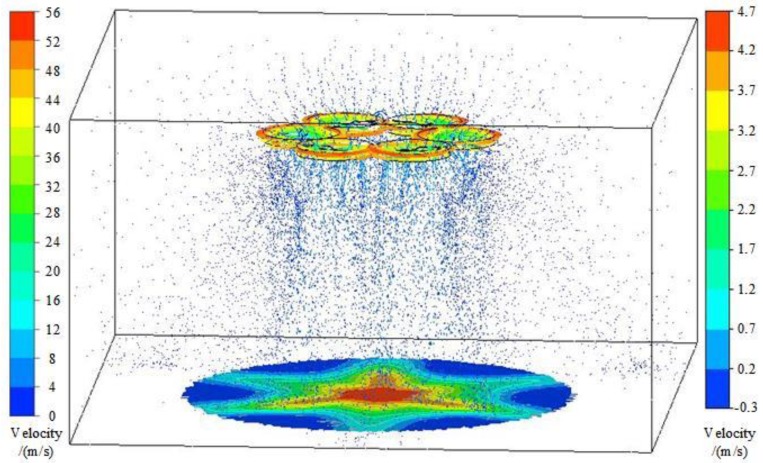
Simulation of the downwash flow field under the rotor.

The simulation distribution of the downwash airflow field under the rotor is shown in Figure 5. The figure shows the airflow velocity trajectory map, the velocity map in the circumferential rotation domain, and the velocity distribution map of the plane where the flexible detection structure acquisition card was located. The airflow velocity trajectory map and the velocity map in the circumferential rotation domain are indicated by the left scale, and the velocity distribution map of the plane where the acquisition card was located is represented by the right scale. The streamline velocity trajectory shows that the width of the wind field positively correlated with the distance from the rotor. The area of contact between the rotor airflow and the crop canopy determines the extent of droplet deposition. At the same time, according to the velocity distribution map of the plane where the flexible detection structure was located, it can be seen that the airflow velocity in the central area decreases gradually to the surrounding area. The simulation results were consistent with the distribution law of the downwash flow field. Moreover, the opposite directions of the adjacent rotors made the lead-out area and the lead-in area of the air flow, and the velocity distribution inconsistent similar to the observation made by Yang et al. [30]. On this basis, the numerical simulation results were applied to the flexible detection structure acquisition card, and the flexible detection structure acquisition system was simulated and optimized.

#### 3.2.2. Flexible Detection Structure Acquisition Card Simulation Analysis and Parameter Determination

**Figure 6:**
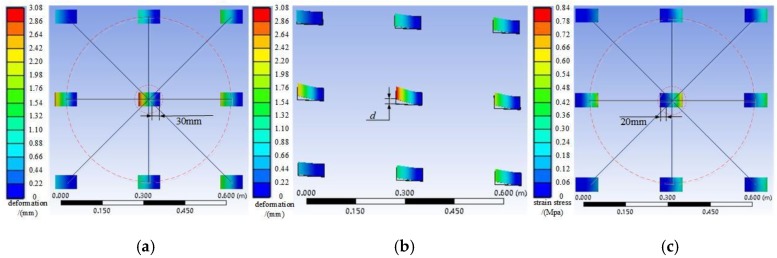
Deformation and strain cloud diagram of a flexible detection structure after flow field action. (**a**) Shape variable cloud; (**b**) Shape change cloud; (**c**) Strain cloud.

It can be seen from the variable cloud diagram (Figure 6a) that the flexible detection structure acquisition piece was arranged in an ‘asterisk’ shape. However, the distribution of the collected shape variables was different after the load was applied by the flow field. It can be seen from the distribution of the shape variables on the entire acquisition surface, there is a certain regularity in the distribution trend. During the process of expanding the middle area to the surrounding area, the maximum shape variable of the flexible detection structure acquisition card was successively decremented. The deformation change cloud diagram is shown in Figure 6b, where Δd is the variation value of the shape variable, which was in accordance with the simulation law of the airflow in Figure 5. There was some regularity in the strain cloud diagram from the fixed end to the far end of the flexible detection structure acquisition card. The fixed end shape variable was the smallest, and the fixed end to the distal end showed obvious deformation phenomenon. It can be seen from the deformation cloud diagram of the whole acquisition surface that when the grating wire length of the navy-blue region was less than 30 mm, there was a small deformation. From this, it can be seen that the grating wire length of the strain gauge should not be too short, and the deformation is not obvious. At the same time, the grating wire length of the strain gauge was too short, which easily caused the error of strain force transmission.

From the strain cloud diagram of Figure 6c, it can be seen that after the load of the flexible detection structure was induced by the flow field, the distribution of the strain force was different. This is consistent with the distribution characteristics of the UAV airflow field and the expectations of this study. During the process of expanding the middle area to the surrounding area, the flexible detecting structure collecting piece shows that the maximum strain force was successively decreased, which also conforms to the simulation law of the air flow in Figure 5. It can be seen from the strain distribution trend of monolithic flexible detection structure acquisition card, the fixed end to the distal shape strain cloud diagram, the fixed end strain force is the largest, the fixed end to the distal end shows the tendency of the strain force to decrease, the distal strain force zero. From the strain surface of the entire collection surface, the navy-blue region is less than 20 mm in length and exhibits a slight strain. It can be seen from the distribution of the strain that the strain gauge had a grating wire length that was not too long, hence the sensitivity was not high enough.

From the variation of Figure 6a and the strain cloud of Figure 6c, it can be seen that the grating wire length of the strain gauge was 50 mm. Under this grating wire length, the bending change was more obvious, and the sensitivity was optimal. In addition, it can be seen from the shape variable diagram and the strain cloud diagram that the force distribution was relatively uniform in the width direction of the flexible detection structure acquisition card. Therefore, in this study, four strain gauges were selected on a single polyester acquisition card, and evenly arranged in the width direction of the polyester acquisition card. This arrangement on the one hand ensures the accuracy of measurement, and on the other hand, four strain gauges can form a full bridge circuit with the highest measurement sensitivity. After the end of the simulation test, the optimization of the parameters was completed, and the experimental research carried out later.

## 4. Experiment Preparation and Test Design

### 4.1. Material and Layout

We used: the data acquisition card of EM9636M; a Dandelion X5 industrial router; waterproof magnifying detection module; SA-50W-12V/4A battery; SE-450-24-450W-24V/18.8A; rip converter (220 V, 1.5 kw); centrifugal air curtain machine (0–15 m·s^−1^); Polypropylene card (5 × 8 cm); BET-6-2020 aluminum bracket (1 × 1 × 1 m^3^); strain gauge (BX120-50AA); Tesco 405i hot-wire anemometer; and a Kestrel 4500 temperature and humidity sensor.

The strain gauge was glued to the flexible structure acquisition card, and three types of bridge (single bridge, half bridge, full bridge) were processed according to the number of strain gauge. The lead end of the acquisition card of the flexible structure acquisition system was fixed at the support card as a fixed end as well as connected to the amplification detection module. The support cards and the aluminum bracket frame are detachable and connected. The outlet of the air curtain machine was parallel to the acquisition surface and located 30 cm above the acquisition surface. The air outlet provided stable airflow, and the wind speed was adjusted by frequency converter. The power supply module, data acquisition card and router were arranged on the base of aluminum bracket frame to avoid interference to wind field detection.

### 4.2. Experiment Design

**Figure 7:**
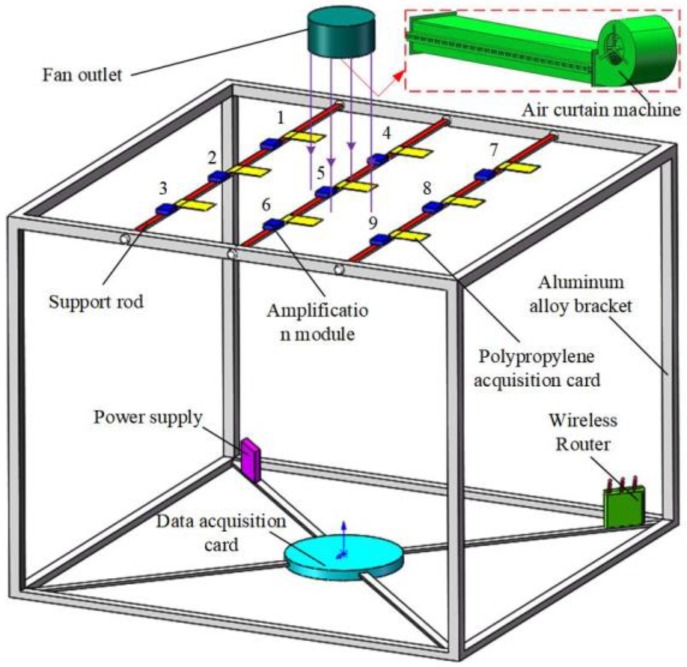
Wind speed test calibration device in lab.

In order to realize the synchronous recording of the wind speed measurement and flexible structure acquisition system, the wind speed detector was placed parallel to one side of the flexible acquisition card. Before the test, the surface of the acquisition card was smoothed, and the amplification detection module was zeroed by the universal voltmeter. At the beginning of the test, the outlet of the air curtain was set parallel to the collecting surface and located 30 cm directly above the collecting surface. The air outlet was adjusted to discharge stable wind speed, which was subsequently adjusted using an inverter. To ensure that the surfaces of the acquisition cards were subjected to similar conditions, the position of the air curtain machine was adjusted accordingly in the test, for each wind speed value (v = 1, 1.5, 2, 2.5...to 10 m·s^−1^), in order to ensure the accuracy of the calibration model between wind speed and output voltage. Then, the upper computer window of LabVIEW 2014 software continuously recorded the indication change of the output voltage in the flexible structure acquisition system for 5 s and the mean value was taken. The whole process was repeated six times. In order to affirm the consistency of the flexible structure acquisition system on the whole acquisition plane, nine acquisition cards of the full-bridge flexible detection cards were tested in parallel at a speed of 4 m·s^−1^. The temperature was 19° ± 0.5° and humidity was 20% ± 0.8%. The experiment layout is shown in Figure 7.

### 4.3. Influence Test and Analysis of Difference in Flexible Structure Acquisition System

The consistency of the flexible structure acquisition system in the whole acquisition plane (the consistency and accuracy of wind speed parameters) is of great significance to the practicability and reliability of the flexible structure acquisition system. Therefore, under a wind speed of 4 m·s^−1^, the performance of nine flexible structure acquisition cards with different bridge forms in the whole acquisition surface were tested in this paper. The test results are listed in Table 1.

In order to compare the uniformity of the output voltage distribution of the nine flexible structure acquisition cards with different bridge forms in the whole acquisition surface, the distribution variation coefficient was taken as the evaluation index.

(10) $$C_{v}=\frac{\sigma }{\bar{u}}\times 100\%,$$

(11) $$\sigma =\sqrt{\frac{\sum_{i=1}^{n}{\left(u_{i}-\bar{u}\right)}^{2}}{n-1}},$$

where, $C_{v}$ is the distribution variation coefficient of output voltage value of the flexible structure acquisition card, %; σ is the standard deviation of output voltage on the collection surface, mv; $u_{i}$ is the voltage values at each sampling point, mv; $\bar{u}$ is the average output voltage on the acquisition surface, mv; n is the number of sampling points of output voltage on the sampling surface, *n* = 9.

According to the results in Table 1, the output voltage variation coefficients of single bridge, half bridge and full bridge were 3.67%, 1.63% and 1.5%, respectively, which implied that the system had a good data acquisition consistency.

### 4.4. Calibration Test and Result Analysis of the Flexible Structure Acquisition System

**Figure 8:**
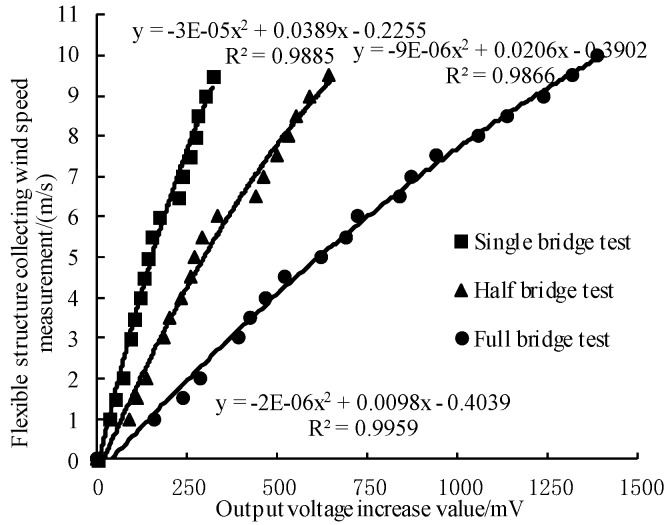
Regression equations for single bridge, half bridge and full bridge circuits.

In this paper, the acquisition cards can produce upward and downward bending deformation. On increasing the output voltage of downward bending the deformation was positive, while on increasing the output voltage of upward bending the deformation was negative. According to the principle of the strain effect, the upward bending was consistent with the downward bending calibration model. To better analyze the correlation between output voltage and measure wind speed, a different bridge type of flexible structure acquisition system, polynomial fitting was used to measure the data. Online acquisition voltage output value Δx was the independent variable and wind speed value collected by sensors Δy was the dependent variable. The result of regression analysis is shown in Figure 8.

The deformation law of the flexible structure acquisition card after wind pressure did not completely obey Hooke’s law. This implied that, the logical relationship of the system did not satisfy the linear relationship. In order to improve the test accuracy and goodness of fit, a one-dimensional quadratic model was considered. Comparing the three curves, under the condition of the same wind speed, the sensitivity was better for the whole bridge type than for the half bridge type for the flexible structure of acquisition system. The three tests before and after the voltage value regression correlation with wind speed was fine. The determination coefficients *R*^2^ for the single bridge, half bridge and bridge circuit were 0.9885, 0.9866 and 0.9959, respectively. In addition, the whole bridge flexible structure acquisition system based on the principle of strain effect can characterize the hoisting situation of UAV downwash airflow field according to its own bending deformation direction [6], which is more suitable for the construction and optimization of the spatial wind field model.

### 4.5. Application Test

**Figure 9:**
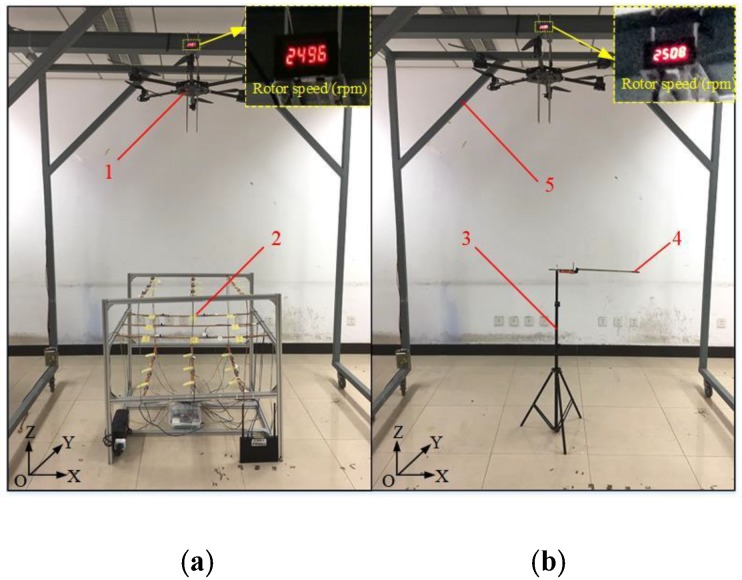
Test arrangement of test platform for rotorcraft UAV and flexible detection structure acquisition system. (**a**) System test platform; (**b**) Wind speed sensor node installation; 1 = Multi-rotor UAV platform; 2 = Flexible detection structure acquisition system equipment; 3 = Triangle bracket; 4 = Wind speed sensor probe; 5 = Mobile bracket.

The application test was carried out on 20 March 2019, in the 5 m × 5 m × 2.6 m area of the Plant Protection Machinery Laboratory in China Agricultural University. In order to ensure the stability of the UAV operation, the multi-rotor UAV used was fixed on the mobile bracket. The complete multi-rotor UAV and the flexible detection structure acquisition system test platform are shown in Figure 9a. According to the experimental platform, a coordinate system was established. The position of the suspension point of the multi-rotor UAV was 0, 0, and 2.6 m, and the position of the center point of the rotor was 0, 0, and 2.35 m, and the aluminum bracket of the flexible detection and collection system was mounted. The support frame was a (1 × 1 × 1 m^3^) cube structure with coordinates (0, 0, 0) at the center point of the bottom surface. During the application test, the rotor speed was obtained by Tachometer (RC41, 0.5‰~1.5‰ rpm, Runchen Company, China). To satisfy the requirements of laboratory safety regulations and aviation plant protection spray environment, a rotor speed of 2500 ± 10 rpm was selected. The multi-rotor plant protection UAV was produced by Shenzhen Jinmingrui Electronics Co., Ltd., and the basic parameters are shown in Table 2.

In order to verify the accuracy of the flexible detection structure acquisition system, and to compare with the wind speed measurement of the system, this paper conducted a parallel test of the downwash airflow velocity. In order to avoid the interference of the artificial measurement on the wind field, the wind speed sensor was fixed on the triangular bracket (height was adjustable, and the distance was retractable). The center position coordinate of the bottom surface of the triangular bracket was 0, 0, 0 against X, Y and Z axis, which can realize each test point of wind speed. The coordinate positions of each test point were consistent with the coordinates of the flexible detection structure acquisition system. The coordinate positions of each test point were consistent with the coordinates of the flexible detection structure acquisition system. The operation steps of the wind speed sensor measurement method were as followed. At each test point, the wind speed sensor wirelessly transmits the three test acquisition data to the mobile APP interface [32] to obtain the wind intensity of the monitored vegetation and records the average value of the wind strength as the air flow speed of the test point. The wind speed sensor node was installed as shown in Figure 9b.

**Figure 10:**
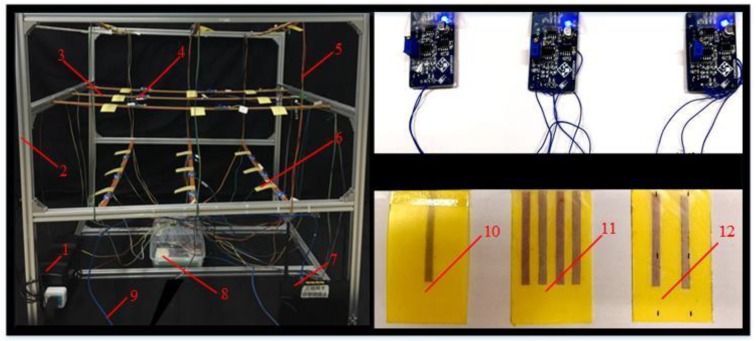
Test layout of test platform for full bridge flexible detection structure acquisition system. 1 = Power supply; 2 = Aluminum bracket; 3 =Support rod; 4 = Waterproof type amplification detection module; 5 = Lead; 6 = Polypropylene acquisition card; 7 = Dandelion X5 industrial grade router; 8 = Waterproof electric control box; 9 = Cable; 10 = Single bridge acquisition system; 11 = Full bridge acquisition system; 12 = Half bridge acquisition system.

In order to test the reliability of the acquisition system, a simulation test of the system wind speed was carried out. The full bridge flexible detection structure acquisition system was placed at three ground heights: 1.0 m, 0.7 m, and 0.4 m, which were designated as upper, middle and lower, respectively. A total of 27 sampling points was set for the aluminum bracket support frame, and nine sampling points were placed for each of the upper, middle and lower layers. The sampling points were numbered in the order of coordinate points. The Z-axis direction was divided into upper, middle, and lower layers. The upper layer was numbered 1–9, and the number of the corresponding position of the upper layer plus the values nine and 18 were the position number values of the middle layer and the lower layer, as shown in Figure 7. On each layer, the flexible structure acquisition cards were arranged in a staggered arrangement, thereby mitigating the interference of the upper layer to the lower layer acquisition card wind field as much as possible, as shown in Figure 10. The acquired analog voltage at each test point was collected and transmitted back to the LabVIEW 2014 interface on the PC side by the wireless router. The operation steps of the system measurement method were as follows; during the test process, each sample point used the waveform diagram of the upper computer software interface to record the wind speed value and the average value collected within 5 s. The collected data was saved and converted into an Excel file.

## 5. Results and Discussion

### 5.1. Testing Results of the Flexible Structure Acquisition System

**Figure 11:**
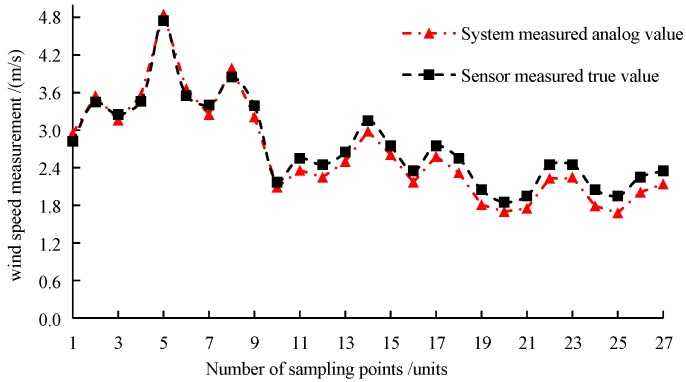
Comparison of wind speed measurement between system analog and sensor measured value at sampling points.

The simulated results and the measured results are shown in Figure 11. In this paper, the measured values are taken as the reference values to calculate the root mean square error (*RMSE*). Equation (12) symbolizes the discrete degree of the simulating value deviating from the measured values. Equation (13) symbolizes the fitting index for analyzing the accuracy of sampling points. Equation (14) symbolizes the confidence index for analyzing the precision of sampling points. Equation (15) symbolizes the maximum relative error of measurement of sampling points [33,34].

(12) $$RMSE=\sqrt{\frac{\sum {}_{i=1}^{N}{\left(X_{obs,i}-X_{model,i}\right)}^{2}}{N}},$$

(13) $$W=1-\frac{\sum {}_{i=1}^{N}{\left(X_{obs,i}-X_{model,i}\right)}^{2}}{\sum {}_{i=1}^{N}{\left(\left|{X^{\prime }}_{obs,i}\right|+\left|{X^{\prime }}_{model,i}\right|\right)}^{2}},$$

(14) $$C=W\times \sqrt{R^{2}},$$

(15) $$e_{\max}=\frac{\left|X_{obs,i}-X_{model,i}\right|}{X_{obs,i}}\times 100\%,$$

where, $X_{model,i}$ is wind speed data (analog value) obtained by using the system method, m·s^−1^; $X_{obs,i}$ is wind speed data (measured value) obtained using a thermal film meter, m·s^−1^; ${X^{\prime }}_{model,i}$ is the deviation from average of analog value; ${X^{\prime }}_{obs,i}$ is the deviation from average of measured value; *C* is the confidence index; $R^{2}$ is the determination coefficient; *N* is the number of samples.

The test samples collected from six replicates were screened, and the size distribution of the downwash airflow field above the ground level of 1.0 m, 0.7 m and 0.4 m was classified. The data obtained by the wind speed sensor was taken as reference. From the testing results, at the height of 1.0 m, the air velocity was between 2.5 m·s^−1^ and 5.0 m·s^−1^ with *RMSE* of 0.1246 m·s^−1^ and *e*_max_ of 5.61%. According to the characteristics of the multi-rotor airflow field, the closer to the rotor surface the greater the air turbulence intensity. The strong pulsating wind speed formed a shock to the flexible acquisition card and caused the fluctuation of output voltage. Although the mean value was obtained under the system calibration method in this paper, there were still some systematic processing errors. At the window of the LabVIEW 2014, the output voltage value within the whole fluctuation was recorded for reference in the future research on the pulsating wind field of multi-rotor UAVs. In Figure 11, at the height of 0.7 m, the wind speed of the downwash airflow field was between 2.0 m·s^−1^ and 3.0 m·s^−1^, *RMSE* of the sampling points was 0.1725 m·s^−1^, and the maximum relative measurement error was 9.91%. At this height, on the one hand, the influence of the system error caused by the pulsating wind field was relatively weak. On the other hand, the error was obviously affected by the shading of the flexible acquisition card and the aluminum bracket. At the height of 0.4 m, the wind speed of the downwash airflow field was between 1.8 m·s^−1^ and 2.5 m·s^−1^. *RMSE* of the sampling points was 0.2238 m·s^−1^ and the maximum relative measurement error was 16.07%. At this height, the sampling point was far away from the rotor, so the system error caused by the pulsating wind field was ignored. The main reason was that the error was obviously influenced by the shielding of flexible acquisition cards and aluminum bracket at the height of 1.0 m and 0.7 m. Secondly, the hot-wire principle of the wind speed sensor in this paper was mainly used for point measurement, while the test system in this paper was surface measurement, which was also a major cause of error. Table 3 shows the regression analysis of the acquisition system in the same layer at different heights from the ground. In conclusion, the fitting index at the height of 1.0 m was higher than those at the height of 0.7 m and 0.4 m, which proved the reliability of the flexible structure acquisition system in this paper and met the application requirements of spray quality measurement of droplets. In order to avoid the errors of 0.7 m and 0.4 m, the flexible acquisition surface can be designed as a lifting platform to avoid the influence of upper acquisition card on the distribution of lower wind field as far as possible.

### 5.2. Analysis of the Wind Field in 3D Shape of the Flexible Structure Acquisition System

**Figure 12:**
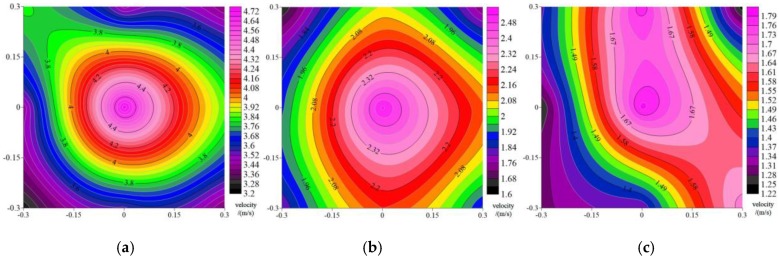
Wind farm data cloud map at heights of 0.4 m, 0.7 m, and 1.0 m in surfer13. (**a**) Speed cloud at 1.0 m altitude; (**b**) Speed cloud at 0.7 m altitude; (**c**) Speed cloud at 0.4 m altitude.

The acquisition system of the flexible structure acquisition system designed in this paper can realize real-time online monitoring of the downwash airflow field of the UAV. The experimental data at the coordinate point can be processed by Surfer 13 software. The contours of the wind field at the height of 0.4 m, 0.7 m and 1.0 m are shown in Figure 12.

From the distribution analysis of contour at a height of 1.0 m, it was known that the distribution of airflow velocity was larger in the center of the space as seen from Figure 12; in the region of the lateral space, the velocity decreases step by step, and the velocity distribution from the central area to the lateral area difference is relatively obvious. This is basically consistent with the UAV simulation flow field distribution results of the flexible structure acquisition system in Figure 5. This also demonstrated the reliability of the simulated results.

According to the distribution analysis of the contour at the height of 0.7 m, the difference in airflow velocity distribution within the whole region was relatively small in Figure 12b compared with Figure 12a, and the maximum airflow velocity gets smaller.

According to the distribution analysis of the contour at the height of 0.4 m, the distribution of air velocity in the whole region had the smallest and most uniform difference in Figure 12c, and the overall distribution characteristics was obviously different from those in Figure 12a,b. In essence, the wind field features were irregular.

### 5.3. Analysis and Discussion on 3D Shape of Wind Field

In this paper, the flexible structure acquisition system adopted 3 × 3 matrix configuration, which can invert the UAV flow field distribution and boundary information [35]. However, due to the lack of collecting cards on the sample surface, the air flow field test was not accurate. As is allowable by space, more sampling cards should be put in the future research, which aims to make the downwash air flow field distribution more accurate. The impeller anemometer has a large shape and has a large interference to the wind field measurement which affects the accuracy of the wind speed measurement. Comparatively, the proposed system has the advantage of wind pressure conversion, the cost is low, there is no inertia delay, and the sensitivity is high. In addition, the system can timely adjust the operating parameters of the UAV according to the wind pressure and growth situation of different crops in the growing period, so as to meet the application requirements of obtaining wind field parameter information in time during the operation of the UAV.

## 6. Conclusions

(1) In this paper, the regression relationship and calculation equations between the UAV downwash air flow field and the parameters of the acquisition system of the flexible structure acquisition system were derived theoretically. Meanwhile, the wind speed test in Z direction of the airflow field was carried out to further prove the feasibility of using the principle of strain effect to measure the downwash airflow field. 

(2) The length and layout of strain gauge gratings in the flexible structure acquisition system were studied. By ANSYS 16.0 finite element simulation software, a coupling model was established to simulate and analyze the strain characteristics of strain gauge gratings in three different lengths. According to the mutual restriction of strain contours, the length of the wire was finally determined to be 50 mm. In order to ensure the accuracy of measurement and the highest sensitivity of the theoretical full-bridge circuit, in this study, four strain gauges were selected on a single polyester acquisition card, and evenly arranged in the width direction of the polyester acquisition card.

(3) The performances of three typical sensor measurement circuits in the measurement of output voltage sensitivity parameters were compared. Firstly, the theoretical deduction showed that the sensitivity of the bridge measurement circuit was full bridge > half bridge > single bridge, and the differential measurement circuit also had the function of temperature compensation. Secondly, the wind pressure caused deformation of different positions on the acquisition surface. Considering the arrangement uniformity of the flexible acquisition card, the full bridge circuit composed of four strain gauges uniformly arranged on the acquisition card of the flexible structure, this implied that the sensing force range was large. In contrast, single bridge and half bridge had limited range of perception of force in terms of placement. Finally, it was verified from experiments that the voltage output fluctuation range of the full bridge flexible detection structure acquisition system was more obvious than that of the single bridge and half bridge flexible detection structure acquisition system, and the sensitivity was better. In conclusion, the full bridge was more suitable for the construction and optimization of the wind speed model. 

(4) From the calibration and application test results in the lab, the designed flexible structure acquisition system was feasible within the range of 0–10 m·s^−1^. The airflow field could be calibrated in a wide range according to the measurement requirements. Surfer 13 software was adopted to further demonstrate the reliability of the flexible structure acquisition system in this paper, which consisted of the simulated result of the downwash airflow field. Combined with experimental research, the fitting index between the simulated value of each acquisition card and the true value of the sensor was 0.961 and the confidence value was 0.9367 at the height of 1.0 m.

(5) In this paper, using the full-bridge strain effect principle and flexible structure acquisition system can realize real-time measurement of the UAV downwash airflow field. The flexible structure acquisition system had high reliability, and great practical significance to developing wind curtain equipment, testing greenhouse mist spraying equipment and studying the distribution characteristics of wind field (the wind velocity distribution variation coefficient, effective width measurement).

## Figures and Tables

**Table 1:** Parameters of fog machine system.

Acquisition System Number	Single Bridge Voltage Output Value/(mv)	Acquisition System Number	Half Bridge Voltage Output Value/(mv)	Acquisition System Number	Full Bridge Voltage Output Value/(mv)
D1	120	B1	234	Q1	469
D2	126	B2	240	Q2	475
D3	119	B3	235	Q3	480
D4	112	B4	246	Q4	468
D5	116	B5	241	Q5	489
D6	118	B6	239	Q6	475
D7	123	B7	236	Q7	471
D8	125	B8	243	Q8	469
D9	120	B9	241	Q9	481

**Table 2:** Technical parameters of multi-rotor unmanned aerial vehicles (UAVs).

Parameter	Data
Motor model	X4114KV370
Distance between axes (mm)	800
Rotor model	1555
Rotor diameter (mm)	380
Number of rotors (unit)	6
Rotor speed (rpm)	2500

**Table 3:** Regression analysis of the acquisition system in the same layer at different heights from the ground.

Height	*W*	*C*	*RMSE*	*e*_max_
0.4 m	0.648	0.6397	0.2238	16.07%
0.7 m	0.831	0.8219	0.1725	9.91%
1.0 m	0.960	0.9367	0.1246	5.61%
